# Punishment-related memory-guided attention: Neural dynamics of perceptual modulation

**DOI:** 10.1016/j.cortex.2019.01.029

**Published:** 2019-06

**Authors:** Samuel Suárez-Suárez, Socorro Rodríguez Holguín, Fernando Cadaveira, Anna C. Nobre, Sonia Doallo

**Affiliations:** a Department of Clinical Psychology and Psychobiology, University of Santiago de Compostela, Santiago de Compostela, Spain; b Department of Experimental Psychology and Oxford Centre for Human Brain Activity, Department of Psychiatry, Wellcome Centre for Integrative Neuroimaging, University of Oxford, Oxford, United Kingdom

**Keywords:** Long-term memory, Visuospatial attention, Punishment, Event-related potentials

## Abstract

Remembering the outcomes of past experiences allows us to generate future expectations and shape selection in the long-term. A growing number of studies has shown that learned positive reward values impact spatial memory-based attentional biases on perception. However, whether memory-driven attentional biases extend to punishment-related values has received comparatively less attention. Here, we manipulated whether recent spatial contextual memories became associated with successful avoidance of punishment (potential monetary loss). Behavioral and electrophysiological measures were collected from 27 participants during a subsequent memory-based attention task, in which we tested for the effect of punishment avoidance associations. Punishment avoidance significantly amplified effects of spatial contextual memories on visual search processes within natural scenes. Compared to non-associated scenes, contextual memories paired with punishment avoidance lead to faster responses to targets presented at remembered locations. Event-related potentials elicited by target stimuli revealed that acquired motivational value of specific spatial locations, by virtue of their association with past avoidance of punishment, dynamically affected neural signatures of early visual processing (indexed by larger P1 and earlier N1 potentials) and target selection (as indicated by reduced N2pc potentials). The present results extend our understanding of how memory, attention, and punishment-related mechanisms interact to optimize perceptual decision in real world environments.

## 1 Introduction

Previous research has demonstrated the ability of spatial contextual long-term memory (LTM) to guide attention within scenes, and to modulate neural signatures of early perceptual analysis (Summerfield, Rao, Garside, & Nobre, 2011) and selection (Patai, Doallo, & Nobre, 2012) of relevant objects presented at memorized locations. This memory-driven modulation of visual processing engages activity in the frontal-parietal network for visual-spatial orienting in concert with activation in other brain regions implicated in retrieval of object locations within specific contexts (e.g., hippocampus) (Stokes et al., 2012, Summerfield et al., 2006). More recent work by Rosen and colleagues shows that LTM-guided visuospatial attention recruits a network spanning parietal cortical areas (lateral intraparietal sulcus, posterior precuneus and posterior callosal sulcus) and subcortical regions (the caudate head, mediodorsal thalamus and lobule VI/Crus I of cerebellum) (Rosen et al., 2017, Rosen et al., 2016). A growing number of studies have also started addressing the important question of whether and how learned reward values impact spatial memory-based attentional biases on perception. Recent work has shown that reward–outcome associations boost memories and attentional priority of specific spatial locations (Chelazzi et al., 2014, Hickey et al., 2014; see also; Anderson, 2015, Pollmann et al., 2016), and dynamically impact different levels of visual cortical processing of targets presented at previously rewarded locations (Doallo, Patai, & Nobre, 2013). These effects of reward history on attentional priority of space add to accumulating evidence indicating that reward-associations modulate bottom-up and top-down attentional priority of stimuli features and object categories (e.g., Anderson et al., 2011, Della Libera and Chelazzi, 2006, Della Libera and Chelazzi, 2009, Donohue et al., 2016, Harris et al., 2016, Hickey et al., 2010, Hickey et al., 2015, Hickey and Peelen, 2015, Kiss et al., 2009; for reviews see; Anderson, 2013, Chelazzi et al., 2013, Failing and Theeuwes, 2018, Pessoa, 2015, Vuilleumier, 2015).

An important remaining question is whether punishment-related associations also influence memory-driven spatial-attentional biases. In contrast to the increasing number of studies examining positive reward-related effects on hippocampus-dependent memory and visuospatial attention, less is known about whether similar effects are observed when spatial locations acquire motivational value through the association with an aversive outcome (or its avoidance). The complexity of this issue increases if we consider that the question of whether reward and punishment processing rely on a common or on distinct neural systems is still debated (Bissonette et al., 2014, Brooks and Berns, 2013, Liu et al., 2011).

Stimuli that undergo aversive conditioning have been shown to exhibit enhanced processing under challenging conditions (Padmala & Pessoa, 2008), to capture attention automatically during visual search (Schmidt, Belopolsky, & Theeuwes, 2015), and to counteract the attentional blink (Lim, Padmala, & Pessoa, 2009). Growing evidence suggests that stimulus features and objects associated with a monetary loss through associative learning also affect attentional and perceptual processes. Face stimuli associated to the receipt of a monetary loss are more likely to be recognized than other faces in a subsequent attentional blink task (Raymond & O'Brien, 2009) and receive enhanced visual processing in a masked recognition task (O'Brien & Raymond, 2012). Stronger reorienting processes for abrupt-onset (exogenous) colored cues linked to a monetary punishment have also been reported (Bucker & Theeuwes, 2016; but see; Rutherford, O'Brien, & Raymond, 2010), as well as increased attentional capture by colored singleton distractors associated to evasion of a monetary loss in a visual search task (Wentura, Muller, & Rothermund, 2014). Recent data reveal that perceptual features associated to receipt of punishment boost primary visual cortex responses (as reflected by the higher amplitude of the C1 potential), relative to reward-associated and neutral ones (Rossi et al., 2017), although evidence for an enhanced representation in visual ventral areas for gain- versus loss-associated stimuli has been found for symbol cues during an economic decision-making task (San Martín, Appelbaum, Huettel, & Woldorff, 2014) and for object categories presented in images of natural scenes (Barbaro, Peelen, & Hickey, 2017).

Though evidence for modulatory effects of punishment associations in visual perceptual and attentional tasks, whether it can be observed for specific locations in cluttered naturalistic contexts remains an open question; furthermore, it is unclear whether it has a lasting effect that can modulate LTM-based attention.

Here, we aimed to shed light on whether and how punishment-related mechanisms influence the ability of LTM to guide perception in naturalistic contexts. We used a similar experimental approach as in our previous study (Doallo et al., 2013) to disentangle whether spatial expectations from LTM incorporate value signals specifically related to past avoidance of an aversive outcome to magnify experience-based biases upon perceptual decisions on relevant objects in cluttered scenes. Event-related potentials (ERPs) were recorded to reveal the time course over which punishment-associated memory-guided attention exerts its effects on the ongoing neural activity in visual cortical areas. We analyzed the P1, N1 and N2pc components. These potentials reflect visual processing (P1 and N1) and target selection (N2pc). The P1 potential, a positive deflection peaking about 100 msec after stimulus presentation, is modulated by visuospatial attention and thought to reflect a sensory gain control mechanism within extrastriate visual cortex (Heinze et al., 1994, Hillyard et al., 1998, Martínez et al., 1999). The N1 potential is a negative deflection, subsequent to the P1, which is also modulated by attention. The N1 attention effect is believed to index a top-down modulation of discriminative processing in areas of the ventral visual stream (Hopf et al., 2002, Luck, 1995, Vogel and Luck, 2000). The N2pc is an enhanced negativity at posterior electrode sites contralateral to the location of an attended target, typically emerging 200–300 msec after target onset. It is thought to reflect attentional selection of a target among distracters (Eimer, 1996, Luck and Hillyard, 1994) and is generated in parietal and inferior occipital-temporal cortical areas (Hopf et al., 2000). These ERP components have been previously shown to be modulated by memory-guided orienting (Doallo et al., 2013, Patai et al., 2012, Summerfield et al., 2011) and have also been used to investigate value-based modulation of perceptual processing and attentional selection in other types of tasks (Donohue et al., 2016, Harris et al., 2016, Hickey et al., 2010, Itthipuripat et al., 2015, MacLean and Giesbrecht, 2015; Qi, Zeng, Ding, & Li, 2013).

In the present experiment, participants first performed a learning task during which they learnt the spatial location of a predefined target (a small key) embedded within visual scenes. Punishment-related associations were manipulated by punishing bad performance during the last block of the learning task on a proportion of trials. Twenty-four hours later, they completed an LTM-cued visual search task in which they had to discriminate the presence or absence of target key stimuli within the previously studied scenes while ERPs were recorded. The initial presentation of the scene (without the target) served as a contextual memory-based cue to orient spatial attention in each trial. Contextual scene cues could either have been associated with avoidance of an aversive outcome (i.e., potential monetary loss) or have had no outcome association. Based on our prior work (Doallo et al., 2013), we hypothesized that if experience-dependent attentional biases on perception are influenced by punishment avoidance associations, this would lead, specifically, to (i) enhanced behavioral performance, as revealed by improved reaction times and accuracy, and (ii) modulation of neural signatures of early visual processing (expressed as enhanced amplitudes of the P1 potential and earlier latencies of N1) and target selection (indexed by reduced amplitudes of N2pc).

## 2 Materials and methods

### 2.1 Participants

Thirty-two healthy students from the University of Santiago de Compostela (Galicia, Spain) participated in this study for monetary compensation. Participants completed a questionnaire regarding personal history of neurological and/or psychiatric disease, existing chronic disease and/or current pharmacological treatment. All participants gave written consent. Data from five participants were discarded during EEG preprocessing because of excessive artifacts in their EEG recording. The remaining 27 participants (18 women, age range 19–27, mean age 20.89 ± 2.02) were all right-handed and had normal or corrected-to-normal vision. The study was approved by the Bioethics Committee of the University of Santiago de Compostela.

### 2.2 Experimental procedure

**Fig. 1:**
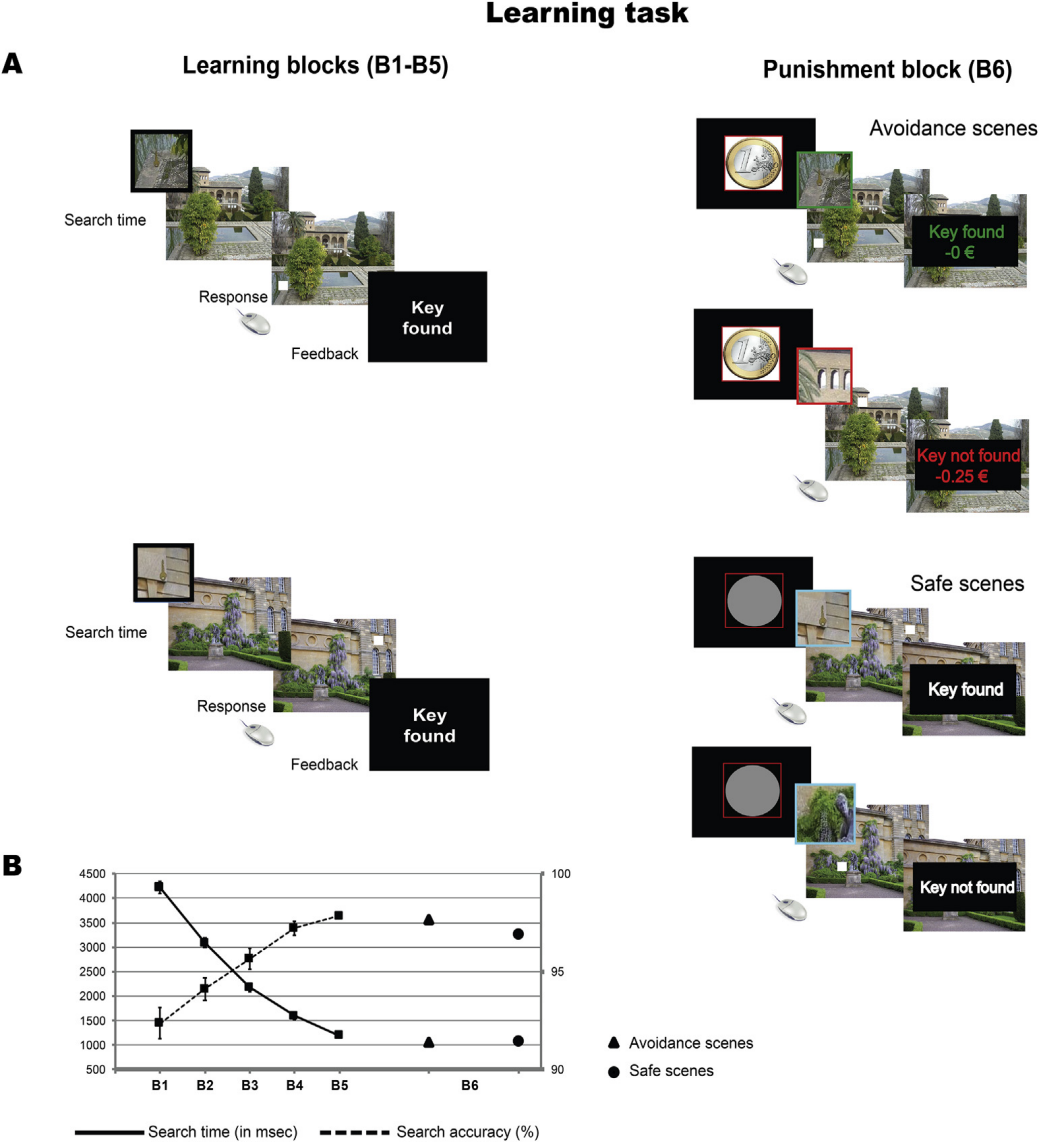
(A) Schematic of the learning task. During this task, participants learnt the spatial location of a target stimulus (a small key) that was embedded within 192 naturalistic scenes repeated in random order over five blocks (left panel). Participants received visual feedback when they correctly identified the location of the key. After the five blocks, participants performed an additional punishment block (right panel), in which the same 192 scenes were presented but one half of them could be followed by a monetary loss (avoidance scenes), cued by a circled coin image, and the other half was followed by no monetary outcome (safe scenes), cued by a grey circle. Visual written feedback after each scene informed participants whether or not they had correctly identified the location of the key or the monetary losses. (B) The graph shows the mean search time and accuracy to detect the presence of the key within each scene across the blocks of the learning task. Results show that participants find more targets and are faster at locating the targets as learning blocks progress. During the final punishment block, there was no difference in performance between avoidance and safe scenes.

**Fig. 2:**
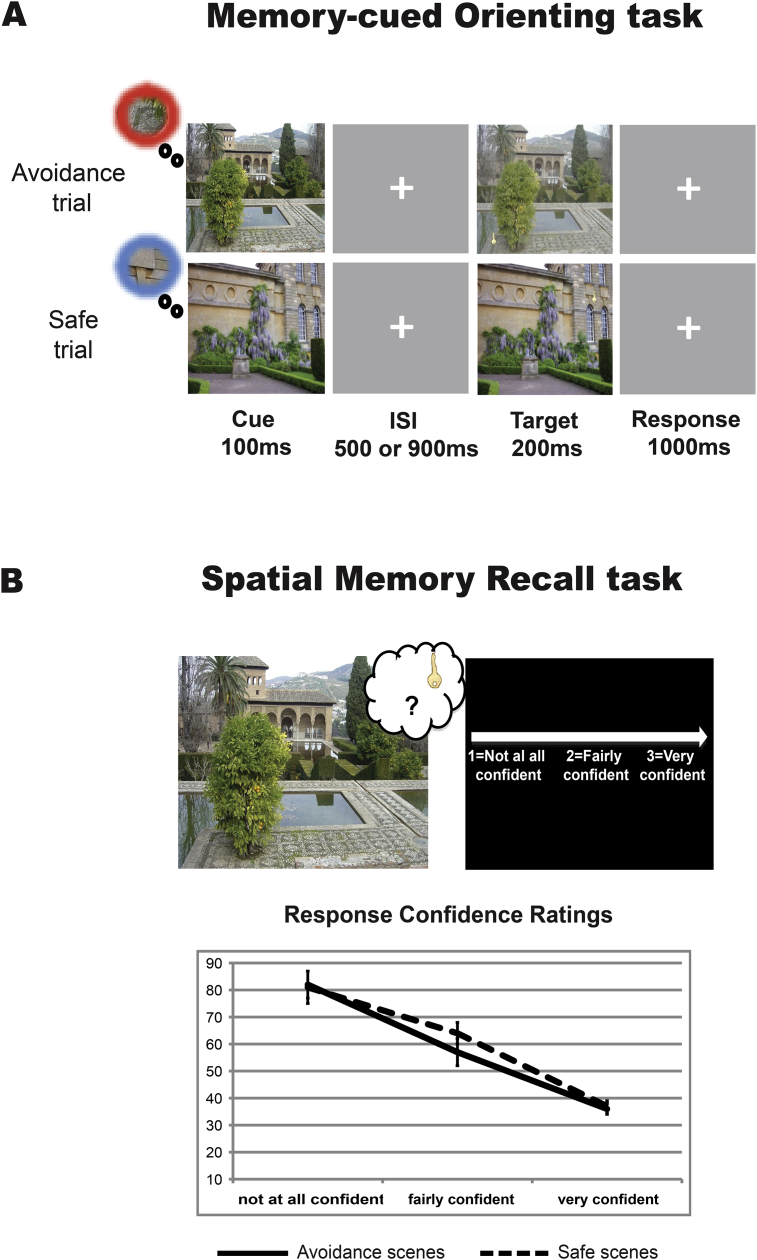
(A) Memory-cued orienting task. Participants had to discriminate the presence or absence of the key target within familiar, studied scenes, making a forced-choice response. The presentation of the scene (without the key) served as the attentional cue. On the top is an avoidance trial where subjects had prior predictive information about where the upcoming target would appear within the scene with a learned punishment avoidance association. On the bottom is a safe trial where subjects had prior predictive information about where the upcoming target would appear without punishment-related association. (B) Design (top figure) and results (bottom figure) of the spatial memory recall task performed immediately after the orienting task. The results showed that the distance (in pixels) between the correct coordinate of the key location and the recalled location decreased systematically as confidence ratings increased.

The task used in this study was a modified version of the experimental design employed in Doallo et al. (2013). There were two phases to the experiment. Participants first performed a learning task (see Fig. 1), followed on the second day by a memory-cued orienting task (in which EEG activity was recorded) (Fig. 2A) and a spatial memory recall task (Fig. 2B).

#### 2.2.1 Stimuli

Two hundred and four digital images of scenes were obtained from the image set used in our previous study (Doallo et al., 2013). A set of 192 scenes was used in experimental trials, and additional 12 scenes were used for familiarization and practice trials. Each scene was prepared, using Matlab (Mathworks, Natick, MA), in two different formats, one for the learning task and one for the orienting task. For counterbalancing purposes, four learning task versions were prepared for each scene with the key (15 x 29 pixels, equivalent to .3° x .7°) placed in one of each of the four visual quadrants, preferably in a hidden location. The assignment of scenes to different experimental conditions, key presence or absence (in the orienting task) and key location within scenes were counterbalanced across participants. For the orienting task, the scenes with keys were remade to include a larger and brighter key (25 x 49 pixels; .6° x 1.1°) in the location of the original key to make it visible within the briefly displayed target scene. Scene stimuli were presented using Presentation (Neurobehavioral Systems, Albany, CA) and subtended 22° x 17° of visual angle at a viewing distance of 100 cm.

#### 2.2.2 Learning task

During the learning task, participants viewed 192 naturalistic scenes repeated in random order over five blocks (Fig. 1A). Participants explored the scenes overtly to search for a small gold key target (present in all the scenes). Once located, they activated the mouse cursor with a left-side mouse click and indicated the location of the key by positioning the cursor on the location of the key and making a second left-sided mouse click. After a response or after the available search time expired, visual written feedback was provided as to whether they had correctly identified the location of the key. Then, the next scene was presented. Allowable search times decreased over blocks: 16–24 sec in Block 1, 12–20 sec in Block 2, 10–18 sec in Blocks 3 and 4, and 8–16 in Block 5. Participants were asked to find as many keys as possible and to memorize their locations. Feedback was also provided at the end of each learning block informing them how many keys they had located during that block.

After the five blocks, participants performed an additional punishment block, in which the same 192 scenes were presented but one half of them could be followed by a monetary loss (avoidance scenes), and the other half was followed by no monetary outcome (safe scenes). Subjects started the experiment with 24€ and were told the total amount they would receive will be determined by their performance in this final block. In avoidance scenes, participants lost .25€ for each key they were unable to find but saved .25€ for each key they found; in safe scenes, performance had no monetary consequence. Participants were cued about the potential consequences of their performance prior to each scene (a circled coin image denoted potential monetary loss and a grey circle denoted no monetary outcome). This minimized the possibility of associating the finding of a key to the avoidance of potential monetary loss in both types of scenes. Again, they received visual written feedback after each scene as a function of their performance. The scene remained onscreen during the presentation of feedback. To ensure that only well-learned key locations were associated to punishment avoidance, the maximum search time was 5 sec in this final block. Avoidance and safe trials were intermixed in a fully randomized fashion.

#### 2.2.3 Memory-cued orienting task

Twenty-four hours later, participants returned to complete a memory-cued orienting task while the EEG was recorded (Fig. 2A). Participants performed 192 trials. They viewed previously studied scenes for a brief exposure and made forced-choice responses, indicating whether a bright gold key was embedded within the scene, and using only covert attention (i.e., maintaining visual fixation on the center of the screen). Each trial began with the presentation (100 msec) of a previously studied scene, which contained no key and acted as valid memory cues (cue scene). After a randomized ISI of 500 or 900 msec, the scene reappeared briefly (200 msec) as the target scene, and participants had to discriminate whether it contained an embedded key. The location of the key target in the previous day's learning task predicted the location where the upcoming target would be presented with 100% validity. Fifty percent of the trials (96) corresponded to the avoidance scenes from the learning task (Avoidance trials: 48 “target present”, 48 “target absent”), and the other half corresponded to the safe scenes (Safe trials; 96 scenes: 48 “target present”, 48 “target absent”). Subjects had a 1000-msec time window to respond after the target scene disappeared. The inter-trial interval varied randomly between 2000 and 3000 msec. Trials were randomly intermixed throughout the task.

Participants carried out a short practice session (12 trials) before the orienting task to ensure they understood the task and could refrain from making eye movements.

#### 2.2.4 Spatial memory recall task

Following the orienting task, participants performed a task measuring explicit memory for the location of the key within each scene (Fig. 2B). Participants viewed all 192 scenes, without any key present. For each scene, they were prompted to indicate whether they could recall the location of the key stimulus via a mouse-button click, and then if possible, the precise location by moving the cursor to the remembered location and clicking again. If they had no memory, they clicked on the center of the screen. Participants were also asked to rate their confidence in their responses after each scene on a 3-point scale by clicking one of the three mouse buttons (1 = *not at all confident*; 2 = *fairly confident*; 3 = *very confident*).

### 2.3 Behavioral statistical analysis

#### 2.3.1 Learning task

The encoding of target–context associations over the course of the learning task, as measured by accuracy (i.e., the mean percentage of keys found in each block) and search time (i.e., the mean search time taken to locate the keys for each block), was assessed by linear contrasts over the five blocks using repeated-measures analyses of variance (ANOVAs). We also conducted a separate ANOVA comparing performance in the punishment (final) block between avoidance and safe scenes that could potentially confound the interpretation of subsequent performance and neural measures on the orienting task. All the subsequent behavioral and ERP analyses used only scenes in which participants had successfully located the target by the final block of the learning task.

#### 2.3.2 Memory-cued orienting task

The effects of punishment-related memory-guided attention on performance were assessed using ANOVAs or paired *t* tests. Reaction times (RTs) to targets and accuracy (i.e., percentage of correct “target-present/target-absent” discriminations) were submitted to a 2 (condition: avoidance, safe) x 2 (target presence: present, absent) ANOVA. The analysis of the orienting task used only scenes in which participants had successfully located the target key by the final block of the learning task (3.41% of the trials were excluded). For RT analysis, only correct trials were used. Trials were excluded if RTs exceed ±3 standard deviations (*SD*; 1.3% of the total trials were excluded). To complement the analysis of RT and accuracy, and to explore the effects of punishment-related memory-guided attention on perceptual sensitivity, we also used a measure from signal detection theory that gives the relationship between the rate of hits to false alarms within each condition [*d’* = *z* (hits) – *z* (false alarms)]. *d’* was compared between avoidance and safe trials using a paired *t* test.

#### 2.3.3 Spatial memory recall task

The distance between the correct coordinate of the key location and the recalled location was computed using only scenes for which the participants had correctly located the key in the learning task. To avoid any contamination effects from re-exposure to target location during the orienting task on the explicit LTM recall, only scenes from “target-absent” trials were analyzed. We used a stringent criterion to test for successful recollection of the key locations—positioning a mouse cursor within a radius of 150 pixels from the target location, equivalent to 3.4°. A repeated-measures ANOVA was used to compare the quality of recollection of the target location, as measured by the distance between the placed cursor and the original key in pixels, across the self-reported confidence levels.

### 2.4 ERP recording and analysis

The EEG was recorded, during the orienting task, using a 64-channels ActiCap (extended 10–20 system). All active electrodes were referred to the nose tip and grounded with an electrode placed at Fpz. The electrooculogram (EOG) was recorded to control for eye movements and blinks. The horizontal and vertical EOG were recorded bipolarly with electrodes placed at the outer canthi and above and below the right eye, respectively. EEG signals were continuously amplified and digitized at a rate of 500 Hz, and filtered with a .01–100 Hz band-pass filter. Data were further low-pass filtered off-line at 40 Hz.

The continuous EEG was segmented into epochs starting 1050 msec before and ending 600 msec after the target scene presentation; this was done to enable removal of any trials with anticipatory saccades. Epochs were baselined from 50 msec before to 50 msec after stimulus presentation. Epochs containing blinks or large saccades (horizontal EOG and vertical EOG exceeding ± 50 μV), excessive noise or drift (a voltage exceeding ± 100 μV at any electrode) were automatically excluded. Epochs were subsequently inspected for smaller saccades, blinks, and drifts and discarded if necessary. Finally, trials with incorrect responses or corresponding to scenes where participants failed to locate the key by the final block of the learning task were excluded from all the further analysis. The Supplementary Figure 2 displays the grand-averaged horizontal EOG waveforms to memory cues directing attention to remembered locations in the left or right visual field in avoidance and safe trials. Averaged horizontal EOG confirms the absence of systematic eye movements toward the memorized target location in response to cue scenes (see also the grand-averaged horizontal EOG activity time-locked to target scenes in Supplementary Figure 3).

Epochs in “target-present” trials were averaged separately according to the main conditions of interest and target side. ERPs from targets located on the right and on the left side of scenes were combined by a procedure preserving the relationship between the side of electrode location and the side of the target (contralateral and ipsilateral). The range of artefact-free target-present trials per average was 20–43 (mean: 31.5) for avoidance trials and 19–42 (mean: 31.3) for safe trials.

To test for modulation of avoidance-associated contextual spatial memory on early visual processing, mean amplitudes of potentials P1 and N1 were measured at contralateral and ipsilateral posterior electrodes (P7/8, P5/6, P3/4, PO9/10, PO7/8, PO3/4, O1/2) during the time windows of 85–110 msec and 130–170 msec, respectively. Peak latency analysis for P1 and N1 were also conducted at these electrode sites in the ranges of 80–150 and 100–200 msec. Time windows for ERPs were selected based on the morphology and timing of the visual potentials averaged across all conditions.

To test how avoidance-associated memory-guided visual search modulated selection of a target within its cluttered naturalistic context, the mean amplitude of the N2pc component was analyzed over parieto-occipital electrodes (PO9/10, PO7/8, PO3/4, O1/2) contralateral and ipsilateral to the side of the target between 190 and 270 msec.

Differences in mean amplitudes and/or peak latencies of potentials were analyzed by repeated-measures ANOVAs with the within-subject factors: condition (avoidance, safe), hemisphere (contralateral, ipsilateral) and electrode location (7 levels for P1 and N1 analyses; 4 levels for the N2pc analysis). The Greenhouse-Geisser correction for non-sphericity was applied when necessary. Post-hoc comparisons were performed using the Bonferroni adjustment for multiple comparisons. The α level was set at *p* < .05. In order to reduce the risk of inflated Type I error rate resulting from testing each hypothesis independently, but to also balance the likelihood of Type II error, we also tested our primary hypotheses using the false discovery rate (FDR) correction (with the Benjamini-Hochberg calculation; Benjamini & Hochberg, 1995). Our 5 a priori expected effects (improved RTs and accuracy, enhanced P1 amplitude, earlier N1 latency and reduced N2pc for avoidance *vs* safe trials of the orienting task) were thus corrected for multiple comparisons. Additional analyses should be considered as exploratory and hypothesis-generating. For all *p* values reported throughout the paper, we provide nominal *p* values and, where appropriate, corrected *p* values using FDR, as well as effect sizes calculated as partial eta-squared (ŋ^2^_p_) values.

### 2.5 Mediation analysis

Mediation analyses were conducted to examine whether modulation of waveform potentials mediated the effect of condition on performance (i.e., whether the benefits in avoidance *vs* safe trials were mediated by modulations of visual cortical processing). We performed separate mediation analyses for repeated-measures designs using the MEMORE macro for SPSS (Montoya & Hayes, 2017), with condition (avoidance *vs* safe) as the independent variable, ERPs (amplitude and/or latency parameters) as the mediators, and behavior (RT, accuracy and *d’*) as the dependent variables. The procedure described by Montoya and Hayes (2017) conceptualizes mediation analysis as a path-analytic framework in which mediation is assessed by a single test of the indirect effect (a*b; i.e., the conjunction of the effect of condition on the potential mediator [path *a*] and the effect of the potential mediator on behavioral performance [path *b*]). The direct effect of condition on behavioral performance that does not operate through the mediator is also calculated (path *c’*). Mediation analyses were conducted using the percentile bootstrap method with 10,000 iterations. The indirect effect was considered statistically significant if the confidence interval (CI 95%) excluded zero.

## 3 Results

### 3.1 Formation of robust LTMs for target locations within natural scenes

Behavioral analysis of the learning task confirmed that participants were able to establish robust memories for the spatial locations at which target stimuli were presented. Over the course of the learning blocks, participants located an increasing number of targets, with increasing speed (Block 1: mean accuracy ± *SEM* = 92.4 ± 1.1%, mean search times ± *SEM* = 4.2 ± .2 sec; Block 5: 97.8 ± .4%, 1.2 ± .1 sec) (Fig. 1B). ANOVAs testing for linear decreases in search times revealed a significant linear contrast over the learning blocks, *F* (1, 26) = 386.64, *p* < .001, ŋ^2^_p_ = .94. A significant linear increase in accuracy over the learning blocks was similarly revealed, *F* (1, 26) = 48.53, *p* < .001, ŋ^2^_p_ = .65. No difference was found between avoidance and safe scenes in the final (sixth) block either in search time [avoidance: 1.1 ± .05; safe: 1.1 ± .05; *F* (1, 26) = .07, *p* = .79, ŋ^2^_p_ = .003] or in accuracy [avoidance: 97.7 ± .5; safe: 96.9 ± .5; *F* (1, 26) = 3.34, *p* = .08, ŋ^2^_p_ = .11].

The recall task performed immediately after the orienting task confirmed that participants retained strong memories of the key locations within the learned scenes on the day after the learning task. On average, participants could explicitly recall the correct locations of targets on 75% of scenes (±2.7 *SEM*). In addition, subjects' confidence ratings varied systematically with the response distance from actual target location, *F* (1.6, 41.6) = 64.96, *p* < .001, ɛ = .8, ŋ^2^_p_ = .71, revealing that higher confidence ratings were associated with more accurate memories [mean distance in pixels ± *SEM*; Rate 1: 81.4 ± 3.9; Rate 2: 60.7 ± 3.5; Rate 3: 36.9 ± 1.6] (see Fig. 2B). Recall did not differ between avoidance-associated and safe key locations [accuracy: 75.9 ± 2.6% *vs* 73.8 ± 3%, *F* (1, 26) = 1.93, *p* = .18, ŋ^2^_p_ = .07; overall distance in pixels: 58.4 ± 3 *vs* 60.9 ± 2.9, *F* (1, 26) = .39, *p* = .54, ŋ^2^_p_ = .02; confidence ratings: 2.67 ± .04 *vs* 2.68 ± .04, *F* (1, 26) = .01, *p* = .92, ŋ^2^_p_ < .001].

### 3.2 One single punishment avoidance-related association enhances the ability of spatial memories to drive visual search in natural scenes

RT and accuracy levels for target-present and target-absent trials in avoidance and safe conditions in the orienting task are summarized in Table 1.

**Table 1:** Reaction time (in msec) and Accuracy (percentage of hits) Values (Mean ± *SEM*) in the memory-cued orienting task.

		Target-Present	Target-Absent
Avoidance trials	RT	649.2 ± 32.5	750.9 ± 32.2
	Accuracy	90.9 ± 1.1	91.8 ± 1.3
Safe trials	RT	654 ± 32.4	764.2 ± 32.9
	Accuracy	89.8 ± 1.2	93.4 ± 1

To test our main hypotheses regarding behavioral performance (improved RT and accuracy), we examined the main effect of condition on these two dependent variables. There was a significant effect on RT, *F* (1, 26) = 6.67, *p* = .016, *p* (FDR corrected) = .048, ŋ^2^_p_ = .204, revealing that target discrimination was faster in avoidance (700.03 ± 31.13) versus safe (709.07 ± 31.22) trials. The main effect of condition on accuracy was not significant [avoidance: .91 ± .01, safe: .92 ± .01; *F* (1, 26) = .09, *p* = .77, *p* (FDR corrected) = .81, ŋ^2^_p_ = .003]. In addition, RT and accuracy ANOVAs showed the following results. Target discrimination was faster in target-present trials [present: 651.58 ± 32.3, absent: 757.53 ± 32.37; *F* (1, 26) = 36.59, *p* < .001, ŋ^2^_p_ = .59], but no significant interaction was found between condition and target presence on RT, *F* (1, 26) = .58, *p* = .45, ŋ^2^_p_ = .02. Analysis of accuracy revealed marginally significant effects for target presence [present: .91 ± .01, absent: .93 ± .01; *F* (1, 26) = 3.93, *p* = .058, ŋ^2^_p_ = .13] and for the interaction between condition and target presence, *F* (1, 26) = 3.89, *p* = .059, ŋ^2^_p_ = .13, which showed a trend for higher accuracy for safe versus avoidance conditions in target absent trials. To test the possibility that RT effects reflected a speed-accuracy trade-off, Pearson's correlation between RT and accuracy effects (calculated by subtracting safe trials from avoidance trials) was performed. A significant negative correlation (*r* = −.48, *p* = .006) indicated that faster responses in avoidance versus safe scenes were associated with higher levels of accuracy.

Finally, the analysis of perceptual sensitivity showed that the *d’* measure was equivalent between avoidance and safe trials [avoidance: 3.27 ± .63, safe: 3.2 ± .61; *t* (26) = .58, *p* = .57].

### 3.3 Punishment avoidance-associated memories modulate target-related neural activity

#### 3.3.1 Early visual processing (P1 and N1 potentials)

**Fig. 3:**
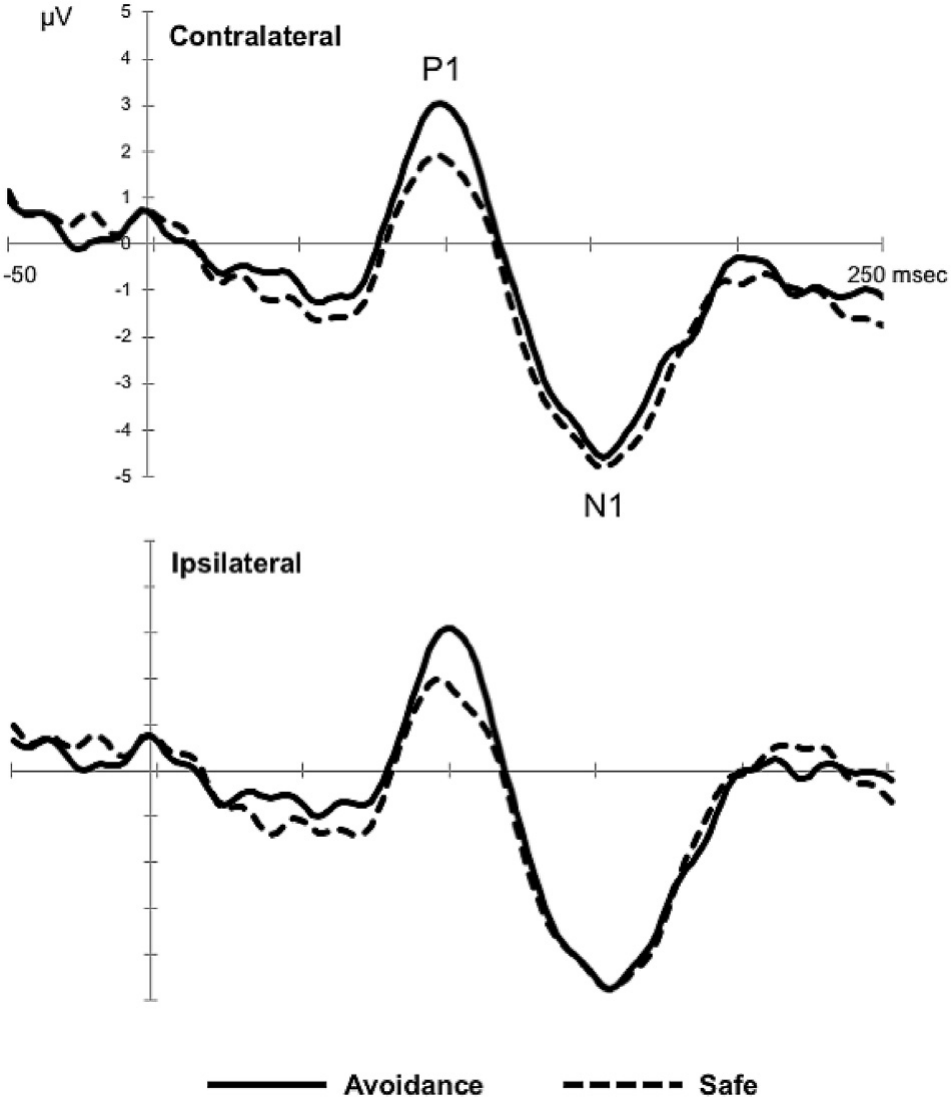
Memory-cued orienting task. Grand-averaged target-locked ERP waveforms (collapsed over electrodes P7/8, P5/6, P3/4, PO9/10, PO7/8, PO3/4, O1/2) for the avoidance and safe conditions.

Target-present scenes elicited the expected visual potentials P1 and N1 over parieto-occipital scalp regions in all conditions (Fig. 3).

As explained in the Introduction section, drawing on our previous work (Doallo et al., 2013; see also; Summerfield et al., 2011), we expected that punishment avoidance-associated memory-based orienting would result in enhanced amplitudes of the P1 potential and earlier latencies of N1. As predicted, there was a significant main effect of condition on P1 amplitude, *F* (1, 26) = 4.31, *p* = .048, ŋ^2^_p_ = .14, revealing that P1 was larger for targets appearing at avoidance-associated remembered locations relative to safe locations. After having identified that one voltage value was outside 3SD from the mean, we verified that the main effect of condition on P1 amplitude remained significant when the outlier was replaced by the series’ mean, *F* (1, 26) = 4.68, *p* = .040, ŋ^2^_p_ = .15. This result, however, did not survive the FDR correction, *p* (FDR corrected) = .067. Regarding the N1 latency, there was no a significant main effect of condition, *F* (1, 26) = 2.45, *p* = .13, *p* (FDR corrected) = .81, ŋ^2^_p_ = .09. The ANOVAs carried out on the P1 amplitude and N1 latency also revealed the following results. A significant main effect of electrode on P1 amplitude, *F* (2.03, 52.66) = 3.73, *p* = .03, *ɛ* = .338, ŋ^2^_p_ = .13, indicated that it was maximal over electrodes P5/6 and PO7/8, but no other main effects or interactions reached significance. The analysis of N1 latency showed that it peaked earlier over the more posterior sites [O1/2, PO3/4; main effect of electrode, *F* (3.09, 80.27) = 10.77, *p* < .001, *ɛ* = .515, ŋ^2^_p_ = .29], and over contralateral (149 ± 2 msec) versus ipsilateral (151 ± 2 msec) sites [main effect of hemisphere: *F* (1, 26) = 4.72, *p* = .039, ŋ^2^_p_ = .15]. There also was a significant hemisphere × condition interaction on N1 latency, *F* (1, 26) = 6.31, *p* = .019, ŋ^2^_p_ = .195, which showed the latencies to be earliest for targets preceded by avoidance-associated memory cues (149 ± 2 msec) than for those preceded by safe memory cues (153 ± 3 msec) over the ipsilateral hemisphere (*p* = .030). The post-hoc comparisons also revealed the effect of hemisphere being significant for safe trials (contralateral *vs* ipsilateral: 150 ± 3 *vs* 153 ± 3 msec; *p* = .009) but not for avoidance trials (149 ± 2 *vs* 149 ± 2 msec; *p* = .708).

The analysis of P1 latency showed earlier latencies at posterior electrodes [O1/2, PO3/4; main effect of electrode: *F* (3.13, 81.29) = 10.28, *p* < .001, *ɛ* = .521, ŋ^2^_p_ = .28], but no significant effects of hemisphere or condition; only subsidiary ANOVAs on a significant 3-way interaction between electrode, hemisphere and condition, *F* (4, 104) = 2.48, *p* = .049, *ɛ* = .667, ŋ^2^_p_ = .09, revealed a significant hemisphere × condition interaction at PO9/10 electrodes, *F* (1, 26) = 5.43, *p* = .028, ŋ^2^_p_ = .17, showing a trend for earlier P1 latencies in avoidance (95 ± 2 msec) versus safe (100 ± 3 msec) trials over ipsilateral sites (*p* = .053).

The analysis of N1 amplitude showed that it was larger at posterior electrodes [O1/2, PO3/4; main effect of electrode, *F* (3.33, 86.59) = 12.83, *p* < .001, *ɛ* = .555, ŋ^2^_p_ = .33], but neither a main effect nor an interaction involving the factor condition were found, indicating that the amplitude of the N1 component was unaffected by type of memory cue.

#### 3.3.2 Target selection (N2pc)

**Fig. 4:**
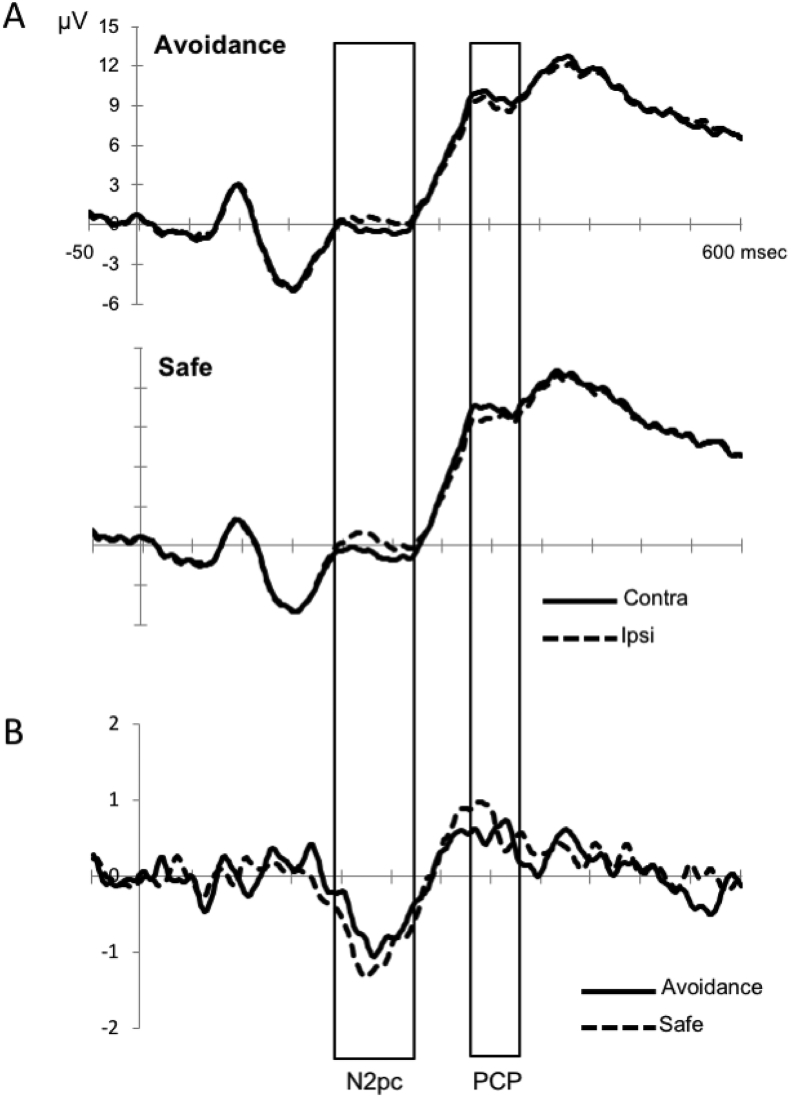
Memory-cued orienting task. (A) Grand-averaged target-locked ERP waveforms (collapsed over electrodes PO9/10, PO7/8, PO3/4, O1/2) in the contralateral and ipsilateral hemispheres for the avoidance and safe conditions. (B) Difference waveforms created by subtracting the ipsilateral from the contralateral target-related ERP waveforms at lateral posterior electrodes for each condition.

The N2pc potential was elicited by target-present scenes at parieto-occipital electrodes contralateral to the side of the target (Fig. 4). The reliability of the N2pc was confirmed by a main effect of hemisphere, *F* (1,26) = 32.28, *p* < .001, ŋ^2^_p_ = .55, on mean amplitudes 190–270 msec after target onset. If as predicted, based on our previous research, N2pc amplitude is modulated by the motivational value of the preceding cue, this should result in an interaction between hemisphere and condition. The predicted two-way interaction was indeed significant, even after FDR correction, *F* (1,26) = 6.23, *p* = .019, *p* (FDR corrected) = .048, ŋ^2^_p_ = .19, indicating that, as expected, the amplitude of N2pc became attenuated by punishment avoidance associated-memory cues. Post-hoc analysis indicated that a significant N2pc was elicited in safe trials (*p* < .001) and, although smaller, was also present in avoidance trials (*p* = .001).

To confirm that the N2pc was related to the selection of the target in its scene context and to rule out that it may have been driven simply by the orienting of spatial attention, we also tested for the presence of the N2pc in target-absent trials. In these cases, there was no significant main effect of hemisphere, *F* (1,26) = .73, *p* = .40, ŋ^2^_p_ = .03, on mean amplitudes 190–270 msec after scene onset, confirming that N2pc was not observed when no target was present.

Visual inspection of the waveforms also revealed a later lateralized effect, following the N2pc with opposite polarity (see Fig. 4), which has been also reported in our previous studies examining LTM-guided visual search in naturalistic scenes (labeled as posterior contralateral positivity, PCP; Doallo et al., 2013, Patai et al., 2012). This effect was an enhanced positivity over posterior contralateral (relative to ipsilateral) scalp locations to the target side in the latency window between 320 and 380 msec poststimulus. An ANOVA analyzing ERP mean amplitudes through this latency window over PO9/10, PO7/8, PO3/4 and O1/2 confirmed the presence of this lateralized effect [main effect of hemisphere: *F* (1,26) = 14.67, *p* < .001, ŋ^2^_p_ = .36], but it was not differentially modulated by punishment avoidance-associated contextual memories [hemisphere x condition: *F* (1,26) = .45, *p* = .508, ŋ^2^_p_ = .02].

### 3.4 Relationship between behavioral and electrophysiological measures

Mediation analyses showed that the P1 amplitude mediated the effect of condition on RT, *ab* = −4.227, *bootSE* = 2.739, CI95% [-10.499, −.102], which was the only indirect effect significantly different from zero (see also Supplementary Figure 1). This result suggests that faster responses in avoidance relative to safe conditions may occur through changes in sensory processing reflected by the P1 potential (i.e., P1 amplitude seems to predict a significant proportion of the co-variation between condition and RT). The complete pattern of results from the mediation analyses is shown in Supplementary Tables 1–3.

## 4 Discussion

The findings of this experiment demonstrate the role of past avoidance of an aversive outcome in magnifying memory-driven attentional biases upon perceptual decisions on relevant objects embedded in cluttered natural scenes. Importantly, our findings revealed the ability of one single punishment-avoidance association in memory to enhance attention and visual search processes in scenes and to modulate ongoing processing in visual cortical areas.

Behavioral results showed that spatial expectations from LTM associated to successful avoidance of punishment conferred behavioral benefits, as revealed by faster responses to targets placed in punishment avoidance- versus safe-related remembered locations. These findings replicate and extend our previous results (Doallo et al., 2013) by showing that punishment-related LTM, similarly to what is observed when positive reward is involved, allows attention to reach the target location more rapidly compared with when memories not associated to motivational values guide spatial orienting. The observed effects in behavior thus indicate that punishment avoidance can bias attention through associations in memory but in the absence of immediate potentially negative consequences (i.e., no punishment was at stake in the visual search task). The present data expand upon the growing behavioral evidence showing that stimuli associated through learning to punishment avoidance (Wentura et al., 2014) or to an actual monetary loss (Bucker & Theeuwes, 2016) can influence attentional deployment and receive facilitated processing (O'Brien & Raymond, 2012). Unlike our prior study manipulating positive rewards, we did not find significant effects on accuracy. Taking into account that there was also a trend for higher accuracy in safe relative to avoidance trials for target absent scenes, it is hard to say, from the present results, if this difference may be attributable to specifically manipulate avoidance-related values, leading to differential behavioral effects than when gains are involved. In this sense, RT effects in the absence of accuracy ones, related to punishment-associated stimuli, have been reported previously (Bucker & Theeuwes, 2016).

The moment-by-moment record of target-related ERPs showed that the behavioral improvement driven by avoidance-related associations was accompanied by modulation of multiple stages of visual processing. Similarly to the pattern of results observed in our previous study, the earliest effect was observed on the P1 potential, an index of attentional gain in extrastriate visual cortex, which showed larger amplitudes at parieto-occipital sites for targets appearing at punishment avoidance-associated remembered locations (relative to safe-related locations), in the absence of effects on the P1 latency. These findings provide evidence that avoidance-associated memory-driven attention biases early perceptual analysis of relevant objects embedded in crowded real-world scenes. Although the main effect of condition on P1 amplitude, which passed a nominal alpha threshold of .05, did not survive FDR correction, the finding that the P1 amplitude modulation significantly mediated the relationship between punishment-related associations in memory and benefits in the reaction time further underscores the significance of this potential in the prioritized processing of stimuli at spatial locations linked to evasion of punishment in the past.

Our findings seem to differ from some recent reports of ERP modulations by acquired positive and negative motivational salience. Bayer et al. (2017) investigated the interplay between reward prospect (i.e., performance-based monetary incentives) during a cued pattern discrimination task on the activity of the primary and extrastriate visual cortex. Motivational relevance conveyed by the cue (related to both reward approach and punishment avoidance) increased the amplitude of the C1 potential (reflecting early perceptual processing in V1) but had no effects at the P1 level. On the other hand, Hammerschmidt, Sennhenn-Reulen, and Schacht (2017) found that neutral faces previously associated with three different monetary outcomes (gain, loss or zero) impacted differently the amplitude of the P1 potential: the facilitated sensory processing of stimuli with associated motivational salience, as reflected by larger P1 amplitudes, was confined to reward-related faces (relative to the neutral, zero-outcome, ones). When addressing the differences between earlier studies and ours, it is important to take into account that findings such as those from Hammerschmidt et al. (2017) indicate a spatially unspecific effect (rather than limited to specific spatial positions) of acquired motivational salience. The differences between the results reported by Bayer et al. (2017) and those of the current study could reflect differential effects of visual spatial cues conveying information about performance-based monetary incentives and contextual memory cues which acquired value in a prior learning experience. Another contributing factor to these differences could be the challenging perceptual conditions in our task. Our target stimuli were embedded within an associated cluttered scene, which may favor prioritization effects (as reflected by higher amplitudes of P1) by which stimuli appearing at spatial locations associated with avoidance of an aversive outcome in past encounters win representation at the expense of other stimuli.

Regarding the N1 potential, an electrophysiological marker of discriminative processes in areas of the ventral visual stream (Hopf et al., 2002, Luck, 1995, Vogel and Luck, 2000), our results showed a significant interaction between condition and hemisphere on its latency, indicating that orienting of spatial attention based on loss avoidance tend to shortened the latency of the N1 component over ipsilateral sites. Although our primary hypothesis testing did not reveal a main effect of condition on N1 latency, this interaction suggests that contextual memories associated to avoidance of punishment are also able to speed up the discrimination of targets appearing at these locations in subsequent encounters, in line with our prior findings (Doallo et al., 2013). This result, however, should be interpreted with caution because it was not subjected to multiple testing correction.

These avoidance-induced ERP modulations agree with neuroimaging findings demonstrating that acquired motivational value modulates representation of stimuli within visual cortical regions (Anderson et al., 2014, Hickey and Peelen, 2015, Hickey and Peelen, 2017), although those studies manipulated positive reward associations. A recent study by Barbaro et al. (2017), in which participants had to detect examples of object categories in naturalistic scenes, found that reward-associated targets were better represented in ventral visual cortex than loss-associated targets (i.e., targets associated to evasion of a greater monetary loss – 150 points – than if not detected – 50 points), thus revealing that the modulation of visual cortical representations was driven by the valence of the outcome association (positive or negative) rather than by the motivational significance (incentive *vs* non-incentive). Their results indicated a selective bias for object categories associated with positive- relative to negative-valence outcomes under conditions in which the monetary punishment, although small, was unavoidable.

Expectations generated from punishment avoidance-associated LTM also resulted in significant amplitude modulations of the ERP marker of target selection, the N2pc, which survived FDR correction for multiple comparisons. The N2pc is an enhanced negative voltage at posterior electrodes contralateral, as compared with ipsilateral, to the side of the target embedded in a visual search array (Eimer, 1996, Hickey et al., 2009, Luck and Hillyard, 1994). It is thought to originate primarily from posterior parietal and occipito-temporal areas (Hopf et al., 2000), and it appears to reflect the spatial layout of a top-down biasing signal that lead to target selection (Kuo, Rao, Lepsien, & Nobre, 2009). The fact that we only observed the N2pc when the target stimulus was present in the scene reinforces the notion that the N2pc is linked to target-selection processes rather than been driving by the spatial guidance of attention (Brignani, Lepsien, & Nobre, 2010). Furthermore, we provide convergent evidence that N2pc can signal the identification of targets embedded within complex backgrounds. This result replicates our previous findings (Patai et al., 2012) showing a N2pc attenuation by LTM-based spatial contextual memory cues, which was interpreted as reflecting the reduced amount of visual analysis and suppression of distracting stimuli required for effective target selection when the location of the target is cued by its previously learned context, which could have preactivated specific memory traces for target/context configurations. This hypothesis was additionally supported by the fact that this N2pc reduction increased when spatial memories were associated to reward (Doallo et al., 2013), consistently with the idea that reward associations increase the ability of spatial expectations from LTM to bias visual processing (see Sawaki, Luck, & Raymond, 2015 for reduced N2pc amplitudes under visual search conditions in which reward at stake was expected to be higher). The present N2pc attenuation for avoidance relative to safe trials agrees with this hypothesis and shows an effect of punishment avoidance-associated LTM on the spatially specific processing of targets. Our results add to a growing body of research showing value-based N2pc modulations. In the context of visual search tasks, N2pc has been shown to be modulated by attentional capture by reward-associated features (Hickey et al., 2010, Qi et al., 2013), even in the absence of awareness induced by object-substitution masking (Harris et al., 2016), and by reward-associated object categories (Donohue et al., 2016). In contrast to the increasing insight gained into the modulation of N2pc by reward associations, the effects of punishment-related associations have been understudied with some exceptions. San Martín et al. (2014) examined N2pc elicited by outcome predicting visual cues in an economic decision-making task in which participants had to learn, by trial-and-error, the reward predicting value of different cues in order to maximize gains and minimize losses. The N2pc was selectively elicited by gain- relative to loss-predicting and neutral cues, which was interpreted as reflecting attentional focusing toward stimuli with reward-predicting value. Their findings thus revealed that loss-minimization was associated with decreased attentional allocation for loss-predicting cues. The different nature of the loss manipulation in San Martín's study – choice-dependent monetary punishment – and ours – a completely incidental loss association with no monetary penalty during the attentional task – makes it difficult to compare the pattern of results directly.

Interestingly, we also replicated our earlier (and unexpected) finding showing a later, spatially specific effect characterized by a lateralized posterior positivity contralateral to the target location (labeled as PCP), which was not significantly modulated by spatial LTM (Patai et al., 2012) or reward (Doallo et al., 2013). We tentatively interpreted this effect following previous ERP studies reporting similar lateralized ERP activity in this latency range during visual search (Hilimire, Mounts, Parks, & Corballis, 2010), and proposed to index additional processing necessary to individuate the target after is identified under conditions of high competition between stimuli in an array.

An important remaining question concerns to the full characterization of the neural system mediating punishment-avoidance memory-guided attention. Based on accumulating evidence, it can be hypothesized that the reported effects of punishment-avoidance associations may be mediated by modulation of activity in brain regions subserving LTM-driven attention. Human studies investigating the extent to which motivation through threat of potential (monetary) punishment influences neural systems subserving LTM have shown that punishment-motivated declarative encoding is mediated by interactions between the hippocampus and the mesolimbic dopamine system, including the ventral tegmental area (VTA) (Shigemune et al., 2014, Wittmann et al., 2013), similarly to what has been reported for reward-motivated declarative encoding (Adcock et al., 2006, Shohamy and Adcock, 2010, Wittmann et al., 2005). Other studies using the threat of a shock as the incentive have reported, however, that punishment-motivated encoding depends on amygdala neuromodulation (Murty, LaBar, & Adcock, 2012), similarly to what has been observed for negative emotional events (LaBar and Cabeza, 2006, Murty et al., 2010).

The effects of punishment avoidance associations on memory-guided attention may be also mediated by modulation of activity in the frontoparietal spatial attention network. Incentive motivation (i.e., prospect of earning an available reward or avoiding a potential punishment) has been shown to influence attention by enhancing neural processing within the spatial orienting network (Engelmann et al., 2009, Small et al., 2005). However, the neural mechanisms involved in the attentional priority of objects and locations linked to motivational value associations are only beginning to be understood. Furthermore, within this body of research, experiments manipulating punishment-related values are underrepresented in comparison to those with positive rewards. Regions of the frontoparietal network, such as the posterior parietal cortex (PPC) and prefrontal cortex (including the frontal eye fields), have been hypothesized as potential substrates of the changes in the attentional priority of specific spatial locations based on learned positive reward associations (see Chelazzi et al., 2014). These regions have been suggested to be particularly important for encoding a priority map of the environment, namely a topographically organized representation of space containing visual salience and/or behavioral relevance information that is important to guide visuospatial attention (Jerde et al., 2012, Mohanty et al., 2008, Ptak, 2012, Serences and Yantis, 2007, Sprague and Serences, 2013). The PPC has also been involved in the attentional processing of reward-associated stimuli (Anderson et al., 2014). Importantly, some studies have shown a role of the PPC in encoding motivational content rather than valence of stimuli. Kahnt, Park, Haynes, and Tobler (2014) have provided evidence that different regions of the PPC encode the value and salience of appetitive and aversive visual cues (i.e., cues predicting the gain or loss of monetary outcomes). Similarly, Barbaro et al. (2017) reported larger activity in this region to reward- and loss-associated target object categories relative to neutral ones. Of interest is also a study by Pollman et al. (2016) examining the neural mechanisms by which reward associations influence contextual cueing effects (i.e., under conditions in which reward is associated via incidental learning to spatial target-distractor configurations), in which demonstrated reward modulations of the dorsal attention network and the retrosplenial cortex (a region known to be engaged in memory retrieval for scenes; Summerfield et al., 2006). Although the contextual cueing paradigm addresses visual search based on implicit memories and, in our study, attention was driven by memories acquired explicitly, the pattern of results of Pollman et al. provides evidence of reward associations effects in brain regions involved in contextual memory-based visual search.

Beyond the frontoparietal network and cortical and subcortical regions involved in memory for visual scenes, other brain areas thought to represent reward value might also be engaged by punishment-related memory-guided attention. Accumulating evidence from electrophysiological recordings and functional magnetic resonance imaging (fMRI) in nonhuman primates suggests an important role for superior colliculus (Griggs, Amita, Gopal, & Hikosaka, 2018) and posterior basal ganglia circuits (caudal-lateral part of substantia nigra pars compacta and the caudate tail) (Ghazizadeh et al., 2018, Kim et al., 2015) in representing stable long-term object value memories (i.e., reward-based LTMs). Recent human positron emission tomography studies have also implicated the dopamine signaling in the human dorsal striatum in processing stimuli with a history of reward (Anderson et al., 2016), which could provide a teaching signal to shape attentional priority (Anderson et al., 2017). It has been proposed that feedback from the dorsal striatum to the visual cortex and superior colliculus may reflect one potential mechanism for signaling value-based attentional priority (Anderson, 2016). The recruitment of regions of the dopaminergic reward system, including the VTA and substantia nigra pars compacta, by reward-associated object categories has also been reported by human fMRI studies (Hickey & Peelen, 2015). Whether punishment-related associations modulate attentional processing through similar neural mechanisms still, however, need to be determined. Given the low spatial resolution of the ERP method, our study is unable to make specific contributions toward detailing the precise brain areas involved in punishment avoidance-associated memory-guided attention.

Finally, the following limitations of this study should be considered. Firstly, although manipulating loss–avoidance associations allowed us to isolate the specific effects of punishment aversion on memory-guided attention, the additional inclusion of positive reward trials in our experimental design would have allowed a more direct contrast, in the same participants, between the impact of associations in memory related to gaining a reward or avoiding a punishment on behavior and neural processing. Future studies systematically comparing, in a within-subjects design, positive reward and punishment associations are needed to allow a clear dissociation between the effects of motivational factors (the presence or absence of incentives) and emotional valence (positive or negative) on LTM-driven visual search. The second limitation concerns the use of multiple ANOVAs and the corresponding risk of Type I error. When addressing this issue, it is important to take into account that the restricted statistical power, mainly due to the characteristics of this type of experimental design, makes necessary to balance the likelihood of Type II errors. We should note that the ecological validity achieved by the use of memories for specific target locations in real scenes comes at a cost. It limits the number of scenes we can use, and prevents us from repeating scenes during the perceptual discrimination task (without possibly compromising the state of the memories being investigated). This consequently reduces the number of trials available and limits the statistical power that can be achieved in other types of designs. To deal with this limitation and balance the likelihood of Type I and Type II errors, we corrected our primary hypotheses for multiple comparisons, as reported throughout this study.

In conclusion, the present study provides new evidence that memory-dependent spatial attentional biases on perception are influenced by punishment avoidance associations, leading to enhanced behavioral performance and modulation of neural signatures of target processing. It also extends our understanding of the role of acquired motivational value in selectively prioritizing specific spatial locations when searching for objects in naturalistic contexts.

## Data statement

We report how we determined our sample size, all data exclusions, all inclusion/exclusion criteria, whether inclusion/exclusion criteria were established prior to data analysis, all manipulations, and all measures in the study. No part of the study procedures or analyses was preregistered prior to the research being undertaken. Due to policy of the research group, public archiving of data and digital study materials will be done at the time our institution makes available an institutional repository for that purpose. Until then, readers seeking access to the data should contact the corresponding author.

## Declarations of interest

None.
