# Supplementary material for: Punishment-related memory-guided attention: Neural dynamics of perceptual modulation
Source: Cortex. 2019 Jun;115:231–45. doi: 10.1016/j.cortex.2019.01.029 (PMC6525146; doi:10.1016/j.cortex.2019.01.029)
Supplement: Multimedia component 1 [file mmc1.docx]

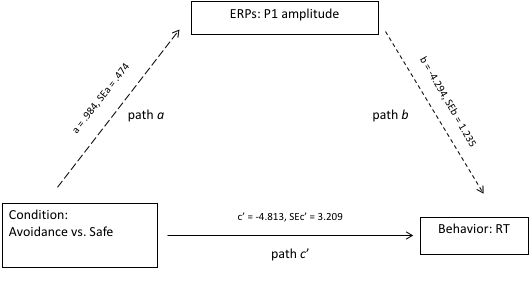
Supplementary data

**Supplementary Figure 1.** Mediation results. The P1 amplitude mediates the relationship between condition (avoidance vs. safe) and reaction time (RT). The indirect effect (*a*b*) is -4.227, *bootSE* = 2.739, CI95% [-10.499, -.102].

**Supplementary Table 1.** Results from the mediation analyses testing whether ERP components mediate condition effects (avoidance vs. safe) on reaction time (RT).

| **RT** | | | | | |
| --- | --- | --- | --- | --- | --- |
| Mediators | | Indirect effect | | Bootstrapping 95% CI | |
|  |  | *ab* | BootSE | Lower | Upper |
| P1 | *Amplitude* | -4.227* | 2.739 | -10.499 | -.102 |
|  | *Latency* | -.145 | 1.107 | -3.094 | 1.376 |
| N1 | *Amplitude* | -.292 | 1.392 | -2.721 | 3.333 |
|  | *Latency* | -1.204 | 1.572 | -5.176 | .991 |
| N2pc | *Amplitude* | -.585 | 2.229 | -4.599 | 4.547 |

**p < 0.05*

**Supplementary Table 2.** Results from the mediation analyses testing whether ERP components mediate condition effects (avoidance vs. safe) on accuracy.

.

| **ACCURACY** | | | | | |
| --- | --- | --- | --- | --- | --- |
| Mediators | | Indirect effect | | Bootstrapping 95% CI | |
|  |  | *ab* | BootSE | Lower | Upper |
| P1 | *Amplitude* | .004 | .004 | -.005 | .012 |
|  | *Latency* | .0001 | .003 | -.004 | .007 |
| N1 | *Amplitude* | -.0003 | .003 | -.009 | .002 |
|  | *Latency* | .002 | .004 | -.002 | .013 |
| N2pc | *Amplitude* | -.003 | .005 | -.016 | .006 |

**Supplementary Table 3.** Results from the mediation analyses testing whether ERP components mediate condition effects (avoidance vs. safe) on *d’* measure.

| ***d’*** | | | | | |
| --- | --- | --- | --- | --- | --- |
| Mediators | | Indirect effect | | Bootstrapping 95% CI | |
|  |  | *ab* | BootSE | Lower | Upper |
| P1 | *Amplitude* | .056 | .064 | -.068 | .196 |
|  | *Latency* | .025 | .056 | -.041 | .182 |
| N1 | *Amplitude* | -.003 | .034 | -.11 | .031 |
|  | *Latency* | .017 | .049 | -.061 | .142 |
| N2pc | *Amplitude* | -.034 | .073 | -.2 | .096 |

.

**
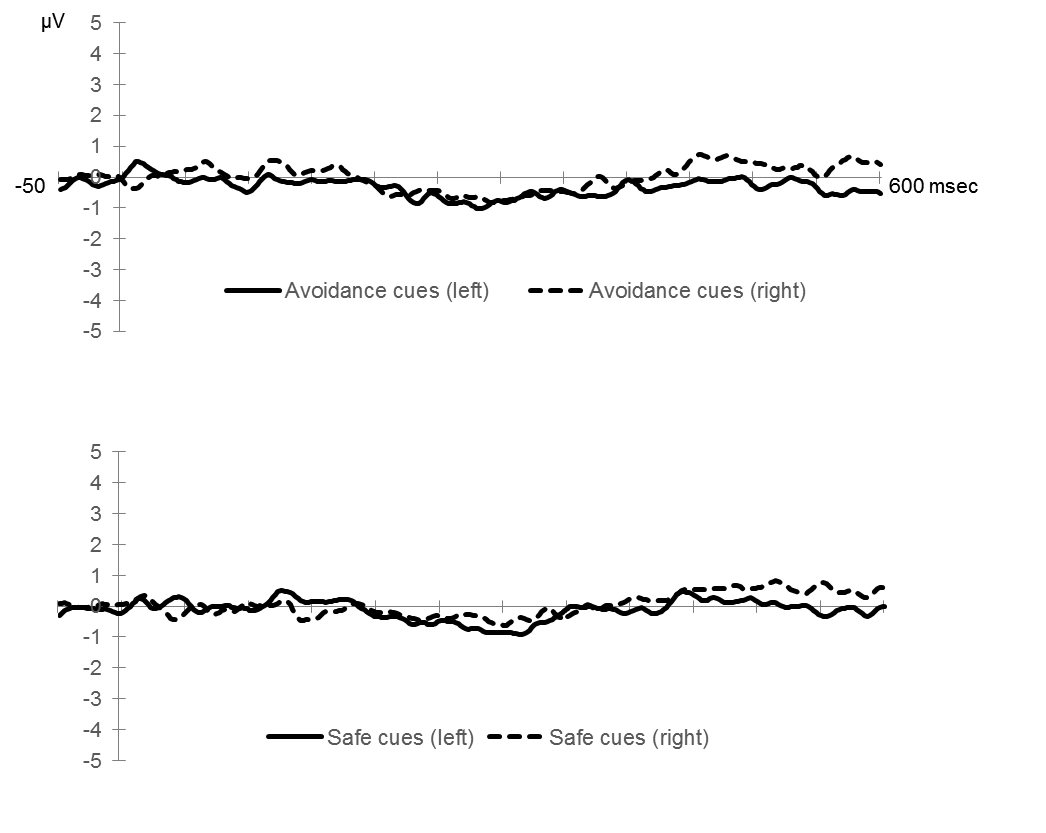
**

**Supplementary Figure 2. Memory-cued orienting task.** Grand-averaged HEOG waveforms time-locked to memory cues directing attention to remembered locations in the right or left visual field, displayed separately for trials with avoidance and safe cues.


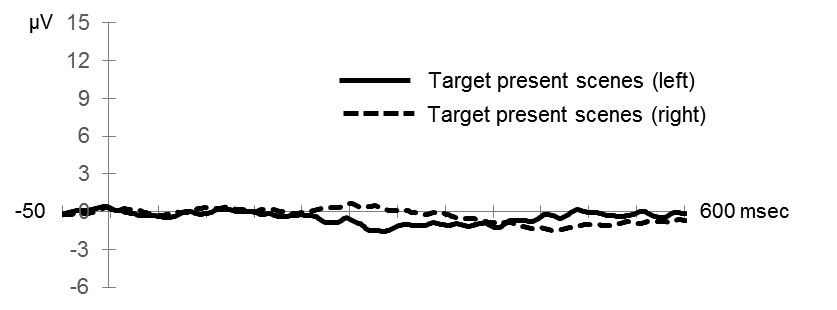


**Supplementary Figure 3. Memory-cued orienting task.** Grand-averaged HEOG waveform time-locked to targets presented at remembered locations in the left or right visual field from avoidance and safe trials (collapsed).
